# The prevalence of hereditary neuromuscular disorders in Northern Norway

**DOI:** 10.1002/brb3.1948

**Published:** 2020-11-13

**Authors:** Kai Ivar Müller, Marijke Van Ghelue, Irene Lund, Christoffer Jonsrud, Kjell Arne Arntzen

**Affiliations:** ^1^ National Neuromuscular Centre Norway and Department of Neurology University Hospital of North Norway Tromsø Norway; ^2^ Department of Clinical Medicine University of Tromsø Tromsø Norway; ^3^ Department of Medical Genetics Division of Child and Adolescent Health University Hospital of North Norway Tromsø Norway

**Keywords:** epidemiology, hereditary, neuromuscular, prevalence

## Abstract

**Aim:**

To investigate the point prevalence of hereditary neuromuscular disorders on January 1, 2020 in Northern Norway.

**Methods:**

From January 1, 1999, until January 1, 2020, we screened medical and genetic hospital records in Northern Norway for hereditary neuromuscular disorders.

**Results:**

We identified 542 patients with a hereditary neuromuscular disorder living in Northern Norway, giving a point prevalence of 111.9/100,000 on January 1, 2020. The prevalence of children (<18 years old) and adults (≥18 years old) were 57.8/100,000 and 125.1/100,000, respectively. Inherited neuropathies had a prevalence of 38.8/100,000. Charcot–Marie–Tooth and hereditary neuropathy with liability to pressure palsies had a prevalence of 29.9/100,000 and 8.3/100,000, respectively. We calculated a prevalence of 3.7/100,000 for spinal muscular atrophies and 2.4/100,000 for Kennedy disease. Inherited myopathies were found in 67.7/100,000. Among these, we registered 13.4/100,000 myotonic dystrophy type 1, 6.8/100,000 myotonic dystrophy type 2, 7.3/100,000 Duchenne muscular dystrophy, 1.6/100,000 Becker muscular dystrophy, 3.7/100,000 facioscapulohumeral muscular dystrophy, 12.8/100,000 limb‐girdle muscular dystrophy, 2.5/100,000 hypokalemic periodic paralysis and 11.4/100,000 myotonia congenita.

**Conclusion:**

Our total prevalence was higher than previously hypothesized in European population‐based studies. The prevalence was especially high for myotonia congenita and limb‐girdle muscular dystrophy. The prevalence of Charcot–Marie–Tooth polyneuropathy was higher than in most European studies, but lower than previously reported in epidemiological studies in other regions of Norway.

## Graphical abstract

Prevalence study of hereditary neuromuscular disorders (HNMD) in Northern Norway based on medical journals, a national Norwegian registry, and medical genetics data. Results showed a high prevalence of HNMD (111.9/100,000) in Northern Norway. The prevalence of Myotonia Congenita and FKRP‐related Limb‐Girdle Muscular Dystrophy R9 might be the highest worldwide.

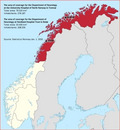

## 1 INTRODUCTION

Hereditary neuromuscular disorders (HNMD) are a heterogeneous group of diseases affecting muscles, neuromuscular junctions, motor neuron cell bodies and peripheral nerves. These disorders are rare, but collectively the influence on health care is noteworthy. Many patients with HNMD need extensive healthcare services. Due to the lack of knowledge of HNMD among healthcare providers, follow‐up and national patient management programs are lacking for most of the subcategories. Consequently, health care differs in various regions.

Worldwide, the total prevalence of HNMD varies with different eras, areas and populations, but studies rarely include all the inherited neuromuscular disorders in all age groups (Hughes et al., 1996; Theadom, Rodrigues, et al., 2019; Theadom, Roxburgh, et al., 2019). Apart from historical studies (Emery, 1991; Hughes et al., 1996), the total prevalence of HNMD in Europe is speculative (Lefter et al., 2017; Norwood et al., 2009). Few Scandinavian studies on the prevalence of inherited neuromuscular disorders exist. A few comprise exclusively children with HNMD (Darin & Tulinius, 2000; Rasmussen et al., 2012), while others are reports on specific HNMD (Braathen et al., 2011; Lindberg & Bjerkne, 2017; Papponen et al., 1999; Stensland et al., 2011; Sveen et al., 2006). Although a recently published study reports on genetic confirmed muscle diseases and spinal muscular atrophy (SMA) in south west Norway, the total prevalence remains uncertain (Husebye et al., 2020).

We need more prevalence studies to plan for diagnostic testing, treatment and follow‐up of HNMD patients. Epidemiological data are necessary to develop clinical management programs and prepare for clinical trials. The increasing molecular diagnostic possibilities and emerging treatment options make awareness of the prevalence even more important. The aim of this study is to estimate the point prevalence (PP) of HNMD and its subcategories in Northern Norway.

## 2 METHODS

### 2.1 Participants, study design and setting

We collected information from the electronic patient hospital records (EPR) of Northern Norway (DIPS ASA, Bodø), the Norwegian registry of hereditary and congenital neuromuscular disorders, and medical genetics records at the University Hospital of North Norway (UNN) in Tromsø. All regional health institutions in Northern Norway (UNN, Finnmark Hospital Trust, Nordland Hospital Trust and Helgeland Hospital Trust) provided the clinical records. The EPR allowed us to screen for specific ICD‐10 diagnoses back to January 1, 1999. Statistics Norway provided information on the population size of Northern Norway at January 1, 2020.

Specified ICD‐10 diagnoses that were reviewed for HNMD in this study are listed in Table 1. In order to identify patients not classified correctly, we made a broad screening (Table 1). The acquired list was merged with the patient list from the Norwegian registry of hereditary and congenital neuromuscular disorders and with the list obtained from the registry at the Medical genetics department at UNN. We used data from the Norwegian National Registry to ensure that all patients were alive and had their residence in Northern Norway. Duplicates identified by checking the Norwegian social security numbers were removed accordingly. Patients included in this study are children and adults with:

- Spinal muscular atrophy type I, II, III IV (SMAI, SMAII, SMAIII, SMAIV) and other inherited spinal muscular atrophies.
- Becker and Duchenne muscular dystrophy (MD), all subcategories of limb‐girdle muscular dystrophy (LGMD) as defined by the 229th ENMC international workshop (Straub et al., 2018), facioscapulohumeral MD type 1 and 2 (FSHD1 and FSHD2), Emery‐Dreifuss MD, myotonic dystrophy type 1 and 2, oculopharyngeal MD, congenital MD and other MD.
- All types of hereditary distal myopathies.
- All types of the congenital myopathies.
- Congenital myotonia (MC) and paramyotonia congenita
- Hyper‐ and hypokalemic periodic paralysis and other hereditary periodic paralysis.
- Congenital myasthenic syndrome.
- All types of primary mitochondrial myopathies and other metabolic myopathies.
- All types of Charcot–Marie–Tooth polyneuropathies (CMT), hereditary neuropathy with liability to pressure palsies (HNPP) and other hereditary neuropathies.

**TABLE 1:** ICD‐10 codes screened from January 1. 1999 – January 1. 2020

ICD‐10	Frequency (*n*)	Percent (%)
G12.0 Infantile spinal muscular atrophy, type I	4	0.7
G12.1 Other inherited spinal muscular atrophy	13	2.4
G12.2 Motor neuron disease	1	0.2
G12.8 Other spinal muscular atrophies and related syndromes	6	1.1
G12.9 Spinal muscular atrophy, unspecified	5	0.9
G60.0 Hereditary motor and sensory neuropathy	149	27.5
G60.1 Refsum's disease	0	0
G60.3 Idiopathic progressive neuropathy	1	0.2
G60.8 Other hereditary and idiopathic neuropathies	21	3.9
G60.9 Hereditary and idiopathic neuropathy	14	2.6
G62.8 Other specified polyneuropathies,	0	0
G62.9 Polyneuropathy, unspecified^a^	5	0.9
G63.3 Polyneuropathy in other endocrine and metabolic diseases	0	0
G63.4 Polyneuropathy in nutritional deficiency	0	0
G63.6 Polyneuropathy in other musculoskeletal disorders	0	0
G63.8 Polyneuropathy in other diseases classified elsewhere	0	0
G70.2 Congenital and developmental myasthenia	1	0.2
G71.0 Muscular dystrophy	109	20.1
G71.1 Myotonic disorders	152	28.1
G71.2 Congenital myopathies	12	2.2
G71.3 Mitochondrial myopathy	7	1.3
G71.8 Other primary disorders of muscles	6	1.1
G71.9 Primary disorder of muscle, unspecified	17	3.1
G72.3 Periodic paralysis	12	2.2
G72.4 Inflammatory myopathy, not elsewhere classified	0	0
G72.8 Other specified myopathies	0	0
G72.9 Myopathy, unspecified	4	0.7
G73.6 Myopathy in metabolic diseases	1	0.2
E74.0 Glycogen storage disease	2	0.4
Total	542	100

Note

Total number of hereditary neuromuscular disorders identified under each ICD‐10 code.

^a^ Only electronic patient records of patients ≤50 years old were screened.

A neurologist (KIM) reviewed all listed EPR journals. According to the EPR, all included patients were given a hereditary neuromuscular diagnosis by either a neurologist, a pediatrician or a geneticist. All included patients had to have findings on neurologic examination. Except for two patients with clinical CMT diagnosis, all patients had abnormalities on either electromyography (EMG), neurography, muscle biopsy or genetic tests that were consistent with the diagnosis of HNMD. Patients with other disorders that could explain the neurological findings, especially those with acquired causes of neuropathy and myopathy, were excluded after thoroughly reviewing the EPR.

Diagnoses were validated independently by another neurologist (KAA). Both neurologists had to concur with each hereditary neuromuscular diagnosis for the patient to be included. Molecular confirmed diagnoses were verified by geneticists (CJ and MVG).

### 2.2 Statistical analysis

The data were analyzed with the Statistical Package for Social Science 26 (SPSS). Confidence intervals of 95% CI were calculated according to Wilsons score interval.

### 2.3 Registration and ethics

The study was approved by the Norwegian National Committee for Medical and Health Research Ethics (NR6859). All data were kept in accordance to the Declaration of Helsinki. We confirm that we have read the Journal's position on issues involved in ethical publication and affirm that this report is consistent with those guidelines. The authors declare no conflict of interest. No financial support for the research/manuscript was received.

## 3 RESULTS

**FIGURE 1:**
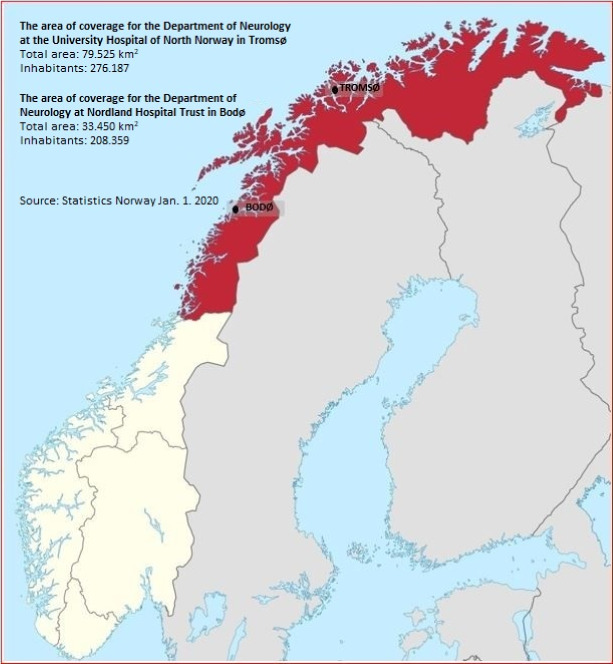
Northern Norway in red color with the two Departments of Neurology in Northern Norway. The departments are located at 69°N in Tromsø city, and at 67°N in Bodø city

Northern Norway encompasses an area of 34.9% of Norway, with 9.0% of the total population (Figure 1). According to Statistics Norway, this area had a total population of 484,546 individuals on January 1, 2020, including 95,182 children <18 years old (whereof 83,879 children <16 years old) and 389,364 adults (≥18 years old). The population consisted of 237,985 (49.1%) females and 246,561 (50.9%) males.

Table 1 provides an overview of the HNMD found in our study according to the ICD‐10 code. Mean age of the HNMD population was 48.8 years (*SD* 21.0) and included 252 (46.5%) females and 290 (53.5%) males. Further, 55 patients were children <18 years old (including 43 children <16 years old) and 487 adults (≥18 years old). The PP of children (<18 years old) and adults (≥18 years old) were 57.8/100,000 (95% CI 44.4–75.2) and 125.1/100,000 (95% CI 114.5–136.7), respectively. The PP of affected children <16 years old was 51.3/100,000 (95% CI 38.1–69.0).

Of the 542 patients with HNMD, 378 (69.7%) had an identified molecular diagnosis, 16 (3.0%) patients were carriers of genetic variants of unknown significance (VUS) and in 148 (27.3%) patients the molecular cause was still unknown (Table 2). Table 2 provides the PP of the main categories of HNMD and their specific clinical diagnosis.

**TABLE 2:** Frequency, prevalence per 100,000 persons, and share of genetically verified patients with hereditary neuromuscular disorders in Northern Norway as of January 1. 2020

	Frequency, *n* (%)	Prevalence (95% CI)	Genetically confirmed, *n* (%)
	(*N* = 542)	111.9 (102.8–121.7)	378 (69.7)
Neuropathies, *n* (%)	188 (34.7)	38.8 (33.6–44.8)	87 (46.3)
Charcot–Marie–Tooth (CMT)	145 (26.8)	29.9 (25.4–35.2)	50 (34.5)
CMT1	39 (7.2)	8.0 (5.9–11.0)	23 (59.0)
CMT1X	9 (1.7)	1.9 (1.0–3.5)	7 (77.8)
CMT2	81 (15.0)	16.7 (13.5–20.8)	12 (14.8)
Intermediate	3 (0.6)	0.6 (0.2–1.8)	0 (0)
CMT4	8 (1.5)	1.7 (0.8–3.3)	8 (100)
HMSN V	1 (0.2)	0.21 (0.04–1.17)	0 (0)
CMT unclassified	4 (0.7)	0.8 (0.3–2.1)	0 (0)
HNPP	40 (7.4)	8.3 (6.1–11.2)	37 (92.5)
Neuralgic amyotrophy	3 (0.6)	0.6 (0.2–1.8)	0 (0)
Spinal Muscular atrophy (SMA)	18 (3.3)	3.7 (2.4–5.9)	12 (66.7)
SMA type I	1 (0.2)	0.21 (0.04–1.17)	1 (100)
SMA type II	1 (0.2)	0.21 (0.04–1.17)	1 (100)
SMA type III	8 (1.5)	0.8 (0.3–2.1)	8 (100.0)
SMA type IV	1 (0.2)	0.21 (0.04–1.17)	1 (100)
SMARD1	1 (0.2)	0.21 (0.04–1.17)	1 (100)
Non‐5q SMA	3 (0.6)	0.6 (0.2–1.8)	0 (0)
SMA unclassified	3 (0.6)	0.6 (0.2–1.8)	0 (0)
Kennedy disease			
Males^a^	6 (1.1)	2.4 (1.1–5.3)	6 (100)
Females^a^	2 (0.4)	0.8 (0.2–3.1)	2 (100)
Myopathies (except channelopathies)	253 (46.7)	52.2 (46.2–59.1)	204 (80.6)
Duchenne muscular dystrophy (MD)^a^	18 (3.3)	7.3 (4.6–11.5)	15 (83.3)
Symptomatic Duchenne carrier^a^	2 (0.4)	0.8 (0.2–0.3.1)	2 (100)
Becker MD^a^	4 (0.7)	1.6 (0.6–4.2)	4 (100)
Facioscapulohumeral MD	18 (3.3)	3.7 (2.4–5.9)	13 (72.2)
Myotonic dystrophy type‐1	65 (12.0)	13.4 (10.5–17.1)	61 (93.9)
Myotonic dystrophy type‐2	33 (5.9)	6.8 (4.8–9.6)	33 (100)
Limb‐Girdle muscular dystrophy (LGMD)	62 (11.4)	12.8 (10.0–16.4)	46 (74.2)
LGMD R1calpain3‐related	4 (0.7)	0.8 (0.3–2.1)	4 (100)
LGMD R9 FKRP‐related	28 (5.2)	5.8 (4.0–8.4)	28 (100)
LGMD R10 titin‐related	1 (0.2)	0.21 (0.04–1.17)	1 (100)
LGMD R12 anoctamin5‐related	6 (1.1)	1.2 (0.6–2.7)	6 (100)
LGMD R22 collagen 6‐related	6 (1.1)	1.2 (0.6–2.7)	1 (16.7)
LGMD D4 calpain3‐related	5 (0.9)	1.0 (0.4–2.4)	5 (100)
LGMD unclassified	11 (2.0)	2.3 (1.3–4.1)	0 (0)
Oculopharyngeal MD	9 (1.7)	1.9 (1.0–3.5)	9 (100)
Myofibrillar MD	4 (0.7)	0.8 (0.3–2.1)	1 (25.0)
Emery‐Dreifuss MD	2 (0.4)	0.4 (0.1–1.5)	2 (100)
Congenital MD	6 (1.1)	1.2 (0.6–2.7)	2 (33.3)
POMT1 MD	1 (0.2)	0.21 (0.04–1.17)	1 (100)
LAMA2 MD	2 (0.4)	0.4 (0.1–1.5)	1 (50)
Unclassified	3 (0.6)	0.6 (0.2–1.8)	0 (0)
Unclassified MD	2 (0.4)	0.4 (0.1–1.5)	0 (0)
Congenital myopathy	3 (0.6)	0.6 (0.2–1.8)	1 (33.3)
Nemalin myopathy	2 (0.4)	0.4 (0.1–1.5)	1 (50)
Multiminicore myopathy	1 (0.2)	0.21 (0.04–1.17)	0 (0)
Rippling muscle disease	1 (0.2)	0.21 (0.04–1.17)	1 (100)
Metabolic myopathies^a^, ^b^	15 (2.8)	3.1 (1.9–5.1)	10 (66.7)
Mitochondrial myopathies	10 (1.9)	2.1 (1.1–3.8)	6 (60.0)
CPO	2 (0.4)	0.4 (0.1–1.5)	1 (50)
MLASA myopathy	2 (0.4)	0.4 (0.1–1.5)	1 (50)
MERRF	1 (0.2)	0.21 (0.04–1.17)	1 (100)
POLG myopathy	1 (0.2)	0.21 (0.04–1.17)	1 (100)
Kearn–Sayre syndrome	1 (0.2)	0.21 (0.04–1.17)	1 (100)
PDHD	1 (0.2)	0.21 (0.04–1.17)	1 (100)
Unclassified	2 (0.4)	0.4 (0.1–1.5)	0 (0)
Glycogen storage disorders	3 (0.6)	0.6 (0.2–1.8)	3 (100)
McArdle disease	3 (0.6)	0.6 (0.2–1.8)	3 (100)
Lipid storage disorders	2 (0.4)	0.4 (0.1–1.5)	1 (50)
MADD	1 (0.2)	0.21 (0.04–1.17)	0 (0)
CPT type‐2	1 (0.2)	0.21 (0.04–1.17)	1 (100)
Distal myopathies	9 (1.7)	1.9 (1.0–3.5)	5 (55.5)
MYH‐7 related myopathy	6 (1.1)	1.2 (0.6–2.7)	3 (50.0)
Welander distal myopathy	1 (0.2)	0.21 (0.04–1.17)	1 (100.0)
GNE myopathy	1 (0.2)	0.21 (0.04–1.17)	1 (100.0)
Distal myopathy unclassified	1 (0.2)	0.21 (0.04–1.17)	0 (0)
Channelopathies	75 (13.9)	15.5 (12.3–19.4)	67 (89.3)
Myotonia Congenita (MC)	55 (10.2)	11.4 (8.7–14.8)	48 (87.3)
Thomsen dominant MC	11 (2.0)	2.3 (1.3–4.1)	10 (90.9)
Becker recessive MC	38 (7.0)	7.8 (5.7–10.8)	38 (100)
Unclassified MC	6 (1.1)	1.2 (0.6–2.7)	0 (0)
Paramyotonia Congenita	7 (1.3)	1.4 (0.7–3.0)	7 (100)
Hypokalemic periodic paralysis	12 (2.2)	2.5 (1.4–4.3)	11 (91.7)
Congenital Myasthenic Syndrome	1 (0.2)	0.21 (0.04–1.17)	1 (100)

Abbreviations: CI, Confidence interval; HMSN, hereditary motor and sensory neuropathy; HNPP, hereditary neuropathy with liability to pressure palsies; SMARD1, Spinal muscular atrophy with respiratory distress type 1.

^a^ Kennedy disease males, Duchenne and Becker prevalence calculated from the share of men in the population. Kennedy disease females and Duchenne carrier calculated from the share of women in the population.

^b^ CPO, chronic progressive external opthalmoplegia; MLASA, lactic acidosis and sideroblastic anemia; MERRF, myoclonic epilepsy with ragged‐red fibers; PDHD, Pyruvate dehydrogenase deficiency; MADD, myoadenylate deaminase deficiency and CPT, Carnitine palmitoyl transferase deficiency.

Of the 188 hereditary neuropathies, 87 patients (46.3%) were genetically confirmed, 7 (3.7%) patients have a VUS and for 94 (50.0%) the molecular cause of disease is still unknown. The 50 CMT patients with an identified genetic cause included: 19 (38.0%) with CMT1A, 4 (8.0%) with CMT1B, 7 (14.0%) with CMT1X, 7 (14.0%) with CMT2A, 3 (6.0%) with CMT2C, 1 (2.0%) with CMT2O, 1 (2.0%) with CMT2I/J and 8 (16.0%) with CMT4. One (0.7%) patient had a CMT diagnosis with central nervous system involvement (HMSN V). The 7 patients with a VUS were 1 CMT1, 1 CMT1X, 1 intermediary CMT and 4 CMT2. In the genetically undetermined CMT patients, 22/95 (23.2%) underwent a Next‐Generation sequencing panel analysis (383 genes listed at https://www.genetikkportalen.no/?act=genpan&katID=19&GpanID=21#popup108), 27/95 (28.4%) underwent multi CMT‐gene sequencing, 2/95 (2.1%) underwent single CMT‐gene sequencing and in 44/95 (46.3%) no genetic CMT‐testing had been ordered. The three patients with neuralgic amyotrophy that were not genetically confirmed, had a positive family history, findings on neurologic examination, EMG and neurography.

The three patients with unclassified SMA have not been genetically tested (Table 2). Two sisters in this category (Table 2) were classified as SMA type III by patient history, neurologic examination, EMG and muscle biopsy. The third unclassified SMA was a patient classified as SMA type IV on the basis of patient history, neurologic examination, EMG and muscle biopsy (Table 2). Three other SMA patients did not have a deletion of exon 7 of the survival motor neuron 1 gene (SMN1) (Table 2). A neuromuscular Next‐Generation Sequencing panel did not reveal any other disease causative variants in two of these patients. The latter non‐5q SMA patient refused further diagnostic testing. Two females (sisters) were diagnosed with Kennedy disease (Table 2). Both inherited an expansion in the androgen receptor (*AR*) gene from each of their parents.

Hereditary myopathies including muscular channelopathies had a PP of 67.7/100,000 (95% CI 60.8–75.4). We found nine patients with inherited myopathies that had a VUS. They included five patients with diagnosis consistent with LGMD R22 collagen six‐related disease/Bethlem, three patients with MYH‐7 related myopathy and one patient with a mitochondrial myopathy, lactic acidosis and sideroblastic anemia (MLASA). Three patients with Duchenne MD were not genetically tested (Table 2), but diagnosed by clinical evaluation and EMG confirmed with muscle biopsy. Eighteen patients were diagnosed with FSHD, including one patient with verified FSHD2, 12 had classical FSHD1 and 5 patients were not genetically verified.

Reviewing the EPRs, seven patients with mitochondrial encephalopathy, lactic acidosis, and stroke‐like episodes (MELAS) were identified, however, only one had been described with muscle symptoms. Therefore, we chose to exclude MELAS from our patient material.

Fifty‐five patients with MC were registered, 45 were living in the northernmost county Finnmark‐Troms. Accordingly, the prevalence of MC in Finnmark‐Troms was 18.5/100,000 (CI 95% 13.8–24.7).

## 4 DISCUSSION

By screening and reviewing EPRs of patients in Northern Norway (Figure 1), genetic records and the Norwegian registry of hereditary and congenital neuromuscular disorders for the last 21 years, we identified 542 patients with HNMD, giving a total prevalence of 111.9/100,000.

A literature search (using PubMed.gov) performed on May 1, 2020 identified few population‐based studies that estimated the total HNMD prevalence in all age groups (Hughes et al., 1996). The prevalence found in the current study is more than three times higher than proposed in 1991, in a historical epidemiological study that combined populations from different parts of the world (33/100,000) (Emery, 1991). Although this was considered a conservative estimate, a similar prevalence was found in 1996 in a study of the HNMD population of Northern Ireland (34.5/100,000) (Hughes et al., 1996). A more recent Irish study analyzing adult HNMD reported a prevalence of 37/100,000 (Lefter et al., 2017). In the latter study the authors state that if they combine the prevalence numbers from their adult population with a prevalence study on children (<16year) in West Sweden, the total HNMD prevalence would probably exceed 100/100,000 (Darin & Tulinius, 2000; Lefter et al., 2017). In a study from Northern England a prevalence of 37/100,000 for hereditary myopathies and 40/100,000 for hereditary neuropathies were established (Norwood et al., 2009). The authors state that by adding prevalence numbers from diagnoses they excluded (mitochondrial and metabolic myopathies, McArdle disease, late Pompe disease and MC), the prevalence of hereditary myopathies would be 50/100,000 (Norwood et al., 2009). Likewise, if all the HNMD would be considered, the prevalence in Northern England would be close to 100/100,000 (Norwood et al., 2009). In line with the previous reasoning, combining results from two recent studies from Spain would hypothetically give a total HNMD prevalence of 102/100,000 (Lousa et al., 2019; Pagola‐Lorz et al., 2019).

Although speculative, these recent total estimates from different European populations are lower than the total prevalence of HNMD in our study, but they correspond well with our prevalence of 52.2/100,000 for hereditary myopathies and 38.8/100,000 for hereditary neuropathies. The higher total prevalence in our study can partly be explained by the high prevalence of MC and LGMD‐R9 FKRP‐related disease. On the other hand, two recently published studies from New Zealand found a total HNMD prevalence of 49/100,000, suggesting ethnic differences (Theadom, Rodrigues, et al., 2019; Theadom, Roxburgh, et al., 2019).

A study from South Norway by Rasmussen and colleagues found that the minimum prevalence of children with HNMD <18 years of age was 36/100,000, which contrasts the 57/100,000 found in West Sweden in children <16 years old (Darin & Tulinius, 2000; Rasmussen et al., 2012). However, the Swedish study used similar methods as the ones applied in the current study and found results within the range of the currently presented results (51.3/100,000) (Darin & Tulinius, 2000). Noticing the similarities, we trust that the total prevalence of HNMD in children (<18 years) in Scandinavia is within the CI of our estimate (44.4–75.2/100,000).

In the current study, the prevalence of CMT neuropathies is in the upper range of that previously reported in Europe (Foley et al., 2012; Lousa et al., 2019), but historically even higher values have been found in two Norwegian populations (Braathen et al., 2011; Skre, 1974). In 1968, Skre found 275 patients with CMT in West Norway, giving a prevalence of 42.3/100,000. However, this study was solely based on patient history and neurological examination (Skre, 1974). In the late ‐90ties, another study reported a CMT prevalence of 82.3/100,000 in one county in the south east of Norway (Braathen et al., 2011). A previous national patient registry search from January 1, 2008–December 31, 2018, estimated that the CMT prevalence could be 34/100.000 (164/486,452) for Northern Norway, equal to the prevalence of 34/100,000 (1817/5,328,000) for all of Norway (unpublished data). A similar prevalence was found in Northern Ostrobothnia in Finland (35/100,000) (Marttila et al., 2017). The findings from the national patient registry and the study from northern Ostrobotnia concurs with the PP in our study (26‐35/100,000).

Surprisingly, in our study CMT2 was the most common CMT, encompassing 55.9% of the total. Among CMT diagnosis, CMT1 predominates in most publications. However, an earlier Norwegian study found an equal distribution between CMT1 and CMT2 diagnoses, whereas a similar higher preponderance of CMT2 has been found in Japan (Braathen et al., 2011; Kurihara et al., 2002). Since few CMT2 patients had been genetically verified in our study, an overestimation of the prevalence is possible. Nevertheless, all CMT2 patients were diagnosed according to patient history, clinical characteristics and neurophysiology. Overall, only about 1/3 of the patients with a CMT diagnosis included in our study were genetically confirmed (Table 2), which is comparable with previous studies (Lefter et al., 2017). The clinical heterogeneity of CMT remains the main challenge in providing a genetically verified CMT diagnosis, and additionally, the genetics of CMT is complex (Juneja et al., 2019).

Worldwide, the most common LGMD is LGMD R1 calpain3‐related disease, but other subcategories may dominate in different ethnic groups and geographical areas (Liu et al., 2019). We found only four patients with recessive calpainopathy, but five with disease caused by the dominant LGMD D4 calpain3‐related disorder (Vissing et al., 2016). Almost half of our LGMD patients had LGMD R9 FKRP‐related disease. Similarly, in the Danish population a high proportion (38%) of LGMD R9 FKRP‐related disease was reported (Sveen et al., 2006). The prevalence of 6/100,000 in our study is three times higher than stated in an early report in the Norwegian population (Stensland et al., 2011), and it is much higher than recently reported in the south west of Norway (0.8/100,000) (Husebye et al., 2020). The prevalence of LGMD R9 FKRP‐related disease in the current study might be the highest known worldwide.

Interestingly, we did not identify any patients with Pompe disease, nor was there any patient with Pompe reported in a study of south west of Norway with a population of roughly 500.000 inhabitants (Husebye et al., 2020). Although we could have missed Pompe disease in the unclassified LGMD and unclassified muscular dystrophy patients (Table 2), this disorder seems to be less frequent in Norway (Husebye et al., 2020). As compared to south west Norway, another less frequent metabolic myopathy in our population was McArdle disease (Husebye et al., 2020). Nevertheless, our prevalence finding of McArdle is comparable to a study of the Irish population (Lefter et al., 2017).

Most studies report a prevalence of MC below 0.5/100,000 in the Caucasian populations (Emery, 1991), and it is extremely rare in other ethnicities (Jou et al., 2004). A recent hospital‐based population study in south west Norway found a higher MC prevalence of 2.8/100,000 (Husebye et al., 2020). However, one of the highest occurrences of MC was found in northern Finland (7.3/100,000), which is previously explained by founder mutations (Papponen et al., 1999). An earlier study from our own region found a minimum prevalence of 9/100,000 (Sun et al., 2001). The higher estimation (11.4/100,000 and even 18.5/100,000 in the northernmost county) in this study is probably due to access to better screening methods in addition to a more thorough review of different medical and genetic EPR. These findings are the highest identified prevalence of MC worldwide. The higher prevalence of MC found in both Norway where it increases at higher latitude and northern Finland is probably due to founder mutations.

Hypokalemic periodic paralysis is an extremely rare disorder. Two European epidemiological studies found a prevalence of less than 0.5/100,000 (Horga et al., 2013; Lefter et al., 2017). The higher occurrence in our population (2.5/100,000) might be because the disease was identified in a few, but large families. We did not identify other hereditary periodic paralysis such as hyperkalemic periodic paralysis and Anderson–Tawil syndrome. Since neither hyperkalemic periodic paralysis nor Anderson–Tawil syndrome was mentioned in the study of the south west Norwegian population, these channelopathies could be less frequent in Norway (Husebye et al., 2020). However, another logic explanation is the cohort size of our study together with the rarity of these disorders. The prevalence of the other reported HNMD in our population (Table 2), were consistent with previously published studies.

Hereditary neuromuscular disorders are rare and accordingly the current study has some weaknesses. The PP can be influenced significantly if a few patients have not been registered, or if an incorrect diagnosis is set. To avoid this, we investigated thoroughly both multiple EPR and registries covering 21 years back in time, and involved two neurologists and two geneticists to secure correct diagnostic procedures. Except from two patients with CMT all diagnoses that were not genetically verified had findings on neurological examination and either EMG/neurography or muscle biopsy that corresponded with an HNMD. Nevertheless, diagnoses that are not genetically confirmed represent a possible weakness in prevalence studies of inherited diseases. We also found 11 patients and six patients with genetically unclassified LGMD and MC, respectively (Table 2), which may accordingly have been misclassified among the different HNMD diagnosis.

However, a strength of the current study is that Northern Norway is sparsely populated and travel distances to hospitals outside the area is extensive. The only neurologic departments are located in Tromsø and Bodø (Figure 1). Due to the huge distances to other neurologic departments and hospitals, it is less likely that patients with HNMD have regularly been followed up in hospitals outside this area. Those few, who travel to other areas, would most likely have been scheduled for local follow‐up management. By using different sources and including all patients seeking specialist care, we do believe to have obtained robust prevalence numbers for HNMD.

Another advantage of the current study is that Norwegian health insurance is equal for the complete population and everybody has the same rights to treatment. However, some patients, especially those with milder disabilities, might not find it necessary to seek healthcare services (e.g., some MC, DM1, CMT and HNPP). The fact that almost a three times higher prevalence of CMT and DM1 has been found in other studies, could imply that the prevalence of less severe manifesting disorders is underestimated in our material (Braathen et al., 2011; Pagola‐Lorz et al., 2019). On the other hand, both regional and ethnic variations of HNMD do exist. Due to the phenotypic variability and the rarity of these disorders, a future comprehensive population‐based epidemiological study based on EPR, the Norwegian registry of hereditary and congenital neuromuscular disorders as well as patient organizations at a national level is warranted.

## 5 CONCLUSION

The total HNMD prevalence in this study was higher than the prevalence of HNMD in hypothetical estimates made from studies across Europe. Among the subcategories, we found high prevalence of MC and LGMD R9‐FKRP‐related disease, and contradicted the previous high prevalence of CMT neuropathies in Norway. The results from this study are important to assess regional differences in prevalence, influence on health care, maintaining and planning high quality and safe diagnostic‐ and treatment regimes, as well as planning cohorts and clinical trials for patients with HNMD.

## CONFLICT OF INTEREST

The authors declare that they have no conflict of interest.

## AUTHOR CONTRIBUTION

Drs. Müller and Arntzen have contributed to the conception and design, acquisition, analysis and interpretation of data. Drs Van Ghelue and Jonsrud contributed to the acquisition, analysis and interpretation of data. MPH Lund contributed to data collection and interpretation. All authors were involved in revising the article and have given approval of the final version.

### Peer Review

The peer review history for this article is available at https://publons.com/publon/10.1002/brb3.1948.

## Data Availability

The data are available from the corresponding author upon reasonable request.
